# Efficacy and Safety of Inclisiran in Adolescents With Genetically Confirmed Homozygous Familial Hypercholesterolemia: Results From the Double-Blind, Placebo-Controlled Part of the ORION-13 Randomized Trial

**DOI:** 10.1161/CIRCULATIONAHA.124.073233

**Published:** 2025-05-20

**Authors:** Albert Wiegman, Amy L. Peterson, Robert A. Hegele, Eric Bruckert, Anja Schweizer, Anastasia Lesogor, Yibo Wang, Joep Defesche

**Affiliations:** Department of Paediatrics (A.W.), Amsterdam University Medical Center, Location Academic Medical Center, University of Amsterdam, The Netherlands.; Department of Human Genetics (J.D.), Amsterdam University Medical Center, Location Academic Medical Center, University of Amsterdam, The Netherlands.; Division of Pediatric Cardiology, Department of Pediatrics, University of Wisconsin School of Medicine and Public Health, Madison (A.L.P.).; Department of Medicine, Schulich School of Medicine and Dentistry, Western University, London, Ontario, Canada (R.A.H.).; Centre de Santé Ellasante, Paris, France (E.B.).; Novartis Pharma AG, Basel, Switzerland (A.S., A.L.).; Beijing Novartis Pharma Co, Ltd, China (Y.W.).

**Keywords:** adolescent, homozygous familial hypercholesterolemia, inclisiran, LDL-C, pediatric

## Abstract

**BACKGROUND:**

Homozygous familial hypercholesterolemia (HoFH) is a genetic disease characterized by high levels of low-density lipoprotein cholesterol (LDL-C) present from birth, leading to early-onset and progressive atherosclerotic cardiovascular disease. Early treatment initiation is crucial for cardiovascular risk reduction; however, many patients do not reach LDL-C treatment goals. Inclisiran, a small interfering RNA targeting hepatic PCSK9 (proprotein convertase subtilisin/kexin type 9), is effective and well tolerated in adult patients with hyperlipidemia; however, it has not yet been studied in pediatric patients.

**METHODS:**

Herein we report results of the 1-year, double-blind, placebo-controlled part of the phase 3 study ORION-13 (Study to Evaluate Efficacy and Safety of Inclisiran in Adolescents With Homozygous Familial Hypercholesterolemia) in adolescents with HoFH. This 2-part multicenter study included 13 patients ≥12 to <18 years of age with a genetic diagnosis of HoFH (excluding LDL [low-density lipoprotein] receptor [*LDLR*] null/null genotypes) and elevated LDL-C levels (>130 mg/dL) on maximally tolerated statin treatment, with or without other lipid-lowering therapies. Eligible patients were randomized 2:1 to receive either 300 mg of inclisiran sodium or placebo, administered on days 1, 90, and 270. The primary end point was the mean percentage change in LDL-C from baseline to day 330.

**RESULTS:**

The mean age of patients was 14.8 years, and mean baseline LDL-C was 272 mg/dL. The placebo-adjusted mean (95% CI) percentage change in LDL-C from baseline to day 330 was −33.3% (−59.2% to −7.3%). Six of 9 (66.7%) inclisiran-treated patients (versus 1 of 4 [25%] on placebo) achieved a >15% reduction in LDL-C, and 5 of 9 (55.6%) inclisiran-treated patients (versus none on placebo) achieved a >20% reduction. The placebo-adjusted mean (95% CI) percentage change in PCSK9 from baseline to day 330 was −60.2% (−79.8% to −40.7%); corresponding changes in apolipoprotein B, non–high-density lipoprotein cholesterol, and total cholesterol were −23.0%, −32.7%, and −27.8%, respectively. No serious adverse events, treatment discontinuations because of adverse events, or deaths occurred. No new safety findings were reported.

**CONCLUSIONS:**

In a 1-year randomized controlled study (part 1 of ORION-13), inclisiran was effective in lowering LDL-C in adolescents with HoFH and was well tolerated. These results support inclisiran as a potentially useful addition for the treatment of adolescents with HoFH and a minimum of LDLR residual activity.

**REGISTRATION:**

URL: https://www.clinicaltrials.gov; Unique identifier: NCT04659863.

**Clinical Perspective**

**What Is New?**

- ORION-13 (Study to Evaluate Efficacy and Safety of Inclisiran in Adolescents With Homozygous Familial Hypercholesterolemia) is the first study to evaluate the efficacy, safety, and tolerability of inclisiran in adolescents with homozygous familial hypercholesterolemia and included patients with residual low-density lipoprotein receptor (LDLR) activity and elevated low-density lipoprotein cholesterol on maximally tolerated statin treatment, with or without other lipid-lowering therapies.
- ORION-13 includes a rigorous placebo-controlled/double-blind design in part 1 (reported here) that is not common among studies in pediatric patients with homozygous familial hypercholesterolemia.
- The placebo-adjusted mean percentage reduction in low-density lipoprotein cholesterol from baseline to day 330 was 33.3%, with a corresponding reduction in PCSK9 (proprotein convertase subtilisin/kexin type 9) of 60.2%.

**What Are the Clinical Implications?**

- In a difficult-to-treat population of adolescents with homozygous familial hypercholesterolemia, and with low recommended low-density lipoprotein cholesterol treatment goals, inclisiran on top of standard-of-care treatment was effective in reducing low-density lipoprotein cholesterol, with a favorable safety and tolerability profile that is consistent with studies in adult patient populations.
- Inclisiran, with an infrequent dosing regimen (twice yearly after the initial and 3-month doses), may be a useful addition to standard-of-care treatment for adolescents with homozygous familial hypercholesterolemia who exhibit at least some LDLR residual activity.

Homozygous familial hypercholesterolemia (HoFH) is a life-threatening genetic disease characterized by high levels of low-density lipoprotein cholesterol (LDL-C) from birth, leading to early-onset and progressive atherosclerotic cardiovascular disease (ASCVD).^1,2^ HoFH is a rare disease, with an estimated prevalence ranging from 1:250 000 to 1:360 000 worldwide.^3,4^ Despite its severity, HoFH often remains undiagnosed or undertreated in childhood and adolescence.^5^

HoFH is caused by pathogenic variants in genes encoding proteins that regulate low-density lipoprotein receptor (LDLR)–mediated clearance of LDL-C, including the *LDLR* gene (85% to 90%), *APOB* encoding apoB (apolipoprotein B; 5% to 10%), and *PCSK9* encoding PCSK9 (proprotein convertase subtilisin/kexin type 9; 1% to 3%).^2^ In addition, there is a rare autosomal recessive form of familial hypercholesterolemia, accounting for <1% of cases, which is caused by biallelic variants in the *LDLRAP1* gene encoding LDLRAP1 (LDLR adaptor protein 1).^6^ The severity of the HoFH phenotype depends on the residual LDLR activity; patients with variants in both alleles causing near or total absence of LDLR activity (ie, *LDLR* null/null) have higher LDL-C levels and poorer clinical prognosis than individuals with variants in 1 or both alleles that only partially reduce LDLR activity (ie, *LDLR* null/defective or *LDLR* defective/defective).^7^ Patients with *LDLR* null/null genotypes often show poor to no response to treatments that upregulate LDLR function.^8–15^

Lowering of LDL-C levels in patients with HoFH reduces the incidence of ASCVD events, and early initiation of treatment is crucial for ASCVD risk reduction.^16,17^ Despite the availability of multiple lipid-lowering therapies (LLTs), with significant progress made in treatment options in recent years, many patients do not reach recommended LDL-C goals,^2,18^ necessitating ongoing research and development of new therapies, especially for young patient populations.

Inclisiran is a first-in-class small interfering RNA that targets hepatic PCSK9 synthesis. Reduced intrahepatic PCSK9 leads to upregulation of LDLRs, thereby increasing LDL-C uptake and thus lowering LDL-C levels in the circulation.^19^ Twice-yearly inclisiran administration (after the initial and 3-month doses) leads to consistent and effective LDL-C lowering and is well tolerated in adult patients with hyperlipidemia.^20–22^

However, inclisiran has not yet been studied in pediatric patients. ORION-13 (Study to Evaluate Efficacy and Safety of Inclisiran in Adolescents With Homozygous Familial Hypercholesterolemia; URL: https://www.clinicaltrials.gov; Unique identifier: NCT04659863) is a phase 3, randomized, 2-part (double-blind inclisiran versus placebo [year 1], followed by open-label inclisiran [year 2]) trial to evaluate the efficacy, safety, and tolerability of inclisiran in adolescents (12 to <18 years of age) with HoFH.^23^ Here, we report the findings from the double-blind, placebo-controlled part 1 of the study.

## METHODS

The data that support the study findings are available from the corresponding author upon reasonable request.

### Study Design and Participants

ORION-13 is a multicenter, randomized, 2-part (double-blind/open-label), phase 3 study, conducted in 9 study centers across 8 countries (Canada, France, Greece, Lebanon, Malaysia, The Netherlands, Türkiye, and the United States). A list of study sites and investigators is provided in Table S1. The objective of the study was to evaluate the efficacy, safety, and tolerability of inclisiran treatment in adolescents with HoFH and elevated LDL-C levels. A detailed study design and the complete list of eligibility criteria have previously been published^23^ and are briefly described herein.

After a 28-day screening/run-in period, the study has 2 sequential parts.^23^ Part 1 was a 1-year double-blind placebo-controlled period in which patients were randomized in a 2:1 ratio to receive either 300 mg of inclisiran sodium (equivalent to 284 mg of inclisiran) subcutaneously or placebo on days 1, 90, and 270. Patients were automatically randomized to a treatment group through interactive response technology, with each patient assigned a randomization number linked to a unique medication number. The randomization scheme was reviewed and approved by an independent group. Patients, study investigators and site staff, and Novartis study staff were blinded to treatment assignment. All lipid, PCSK9, and antidrug antibody results after the screening visit were blinded by the central laboratory. Part 2 consists of a 1-year open-label, single-arm period in which patients already receiving inclisiran continue treatment, and placebo-treated patients from part 1 are transitioned to inclisiran.^23^ The results of the placebo-controlled part 1 of ORION-13 are presented in this report. This report adheres to the Consolidated Standards of Reporting Trials (CONSORT) guidelines.^24^

Male and female adolescents (12 to <18 years of age) were eligible for participation if they had a genetic diagnosis of HoFH (ie, HoFH confirmed by genotyping) and a fasting LDL-C level >130 mg/dL (>3.4 mmol/L) on stable (≥30 days), maximally tolerated (based on the investigator’s discretion) statin treatment with or without other LLTs. Key exclusion criteria were HoFH with documented evidence of a null variant in both *LDLR* alleles, heterozygous familial hypercholesterolemia, active liver disease, secondary hypercholesterolemia (eg, hypothyroidism or nephrotic syndrome), previous treatment with mAbs (monoclonal antibodies) directed towards PCSK9 within 90 days of screening, treatment with mipomersen or lomitapide within 5 months of screening, and recent or planned use of other investigational medicinal products or devices. Patients continued their previous background LLT throughout the study. Patients who were on LDL (low-density lipoprotein) apheresis were allowed to continue the same stable regimen during the study. The apheresis schedule had to allow for apheresis to coincide with each study visit, blood samples had to be drawn immediately before the scheduled apheresis treatment at each applicable study visit, and the study drug had to be administered the same day after completion of apheresis.

### Study End Points

The primary end point was the percentage change in LDL-C from baseline to day 330. Secondary efficacy end points were time-adjusted percentage change in LDL-C from baseline after day 90 and up to day 330, absolute change in LDL-C levels, percentage change in other lipid parameters (including total cholesterol, non-HDL [high-density lipoprotein] cholesterol, and apoB), and PCSK9 from baseline to each assessment time point (including day 330). As an additional secondary end point, safety and tolerability were assessed by treatment-emergent adverse events (AEs) and serious AEs, including their relationship with study drug, and laboratory parameters. In addition, antidrug antibody assessment, growth (height, weight, and body mass index), pubertal development (evaluated using Tanner staging), and hormonal status (estradiol/testosterone, LH [luteinizing hormone], FSH [follicle-stimulating hormone], cortisol, and dehydroepiandrosterone sulfate) were evaluated.

Individual responsiveness of participants, defined as percentage reductions in LDL-C from baseline at day 330, and percentage changes in LDL-C from baseline to day 330 by genotype subgroups were also evaluated as exploratory end points. In addition to the mandatory historical genetic diagnosis, genetic data were also analyzed by a central laboratory (Medpace) when separate informed consent was provided. Adjudication of the central laboratory genetic data into variant groups, including classification of *LDLR* variants (*LDLR* null [<2% residual activity] and *LDLR* defective), was performed by 2 experts from the study’s steering committee. Only adjudicated data were used for analysis. Fasting (≥8 hours) blood samples were drawn for measurements of lipid parameters and all other laboratory parameters by the central laboratory. LDL-C levels were determined using the beta quantification method. The safety data were reviewed by an independently functioning data monitoring committee at regular intervals.

### Statistical Analysis

No sample size calculation was performed; the sample size for this study was selected based on feasibility. Approximately 12 patients were planned to be enrolled, considering the very rare disease population. Efficacy end points were analyzed using the full analysis set, which was defined as all randomized participants except for misrandomized participants who did not receive study drug; participants were analyzed following the intention-to-treat principle (ie, according to the treatment to which they were assigned at randomization). Safety and tolerability end points were analyzed using the safety analysis set, which comprised all participants who received at least one dose of study medication; participants were analyzed according to the treatment received. The primary objective was to evaluate the effect of inclisiran compared with placebo on reducing LDL-C (percentage change) at day 330. Because of the small sample size, all efficacy analyses, including the primary objective, were descriptive, with no formal statistical testing. Mean changes from baseline along with SD by treatment group and differences between treatment groups and 95% CIs were evaluated. The CI was constructed assuming Student *t* distribution and equal variance between the 2 samples. Analysis was performed using SAS (v9.4).

### Ethics Approval

The study was conducted in accordance with the trial protocol, principles of the Declaration of Helsinki, and the International Conference on Harmonization Guidelines for Good Clinical Practice. The institutional review board or independent ethics committee at each study center approved the study protocol, and written informed consent was obtained from each participant’s parent(s) or legal representative(s) and from the participant, if applicable.

### Data Sharing Statement

The sponsor is committed to sharing access to patient-level data and supporting clinical documents from eligible studies with qualified external researchers. These requests are reviewed and approved by an independent review panel based on scientific merit. All data provided are anonymized to respect the privacy of patients who have participated in the trial in line with applicable laws and regulations. The availability of these trial data is in accordance with the criteria and the process described at https://www.clinicalstudydatarequest.com.

## RESULTS

### Baseline Demographic and Clinical Characteristics

**Figure 1.**
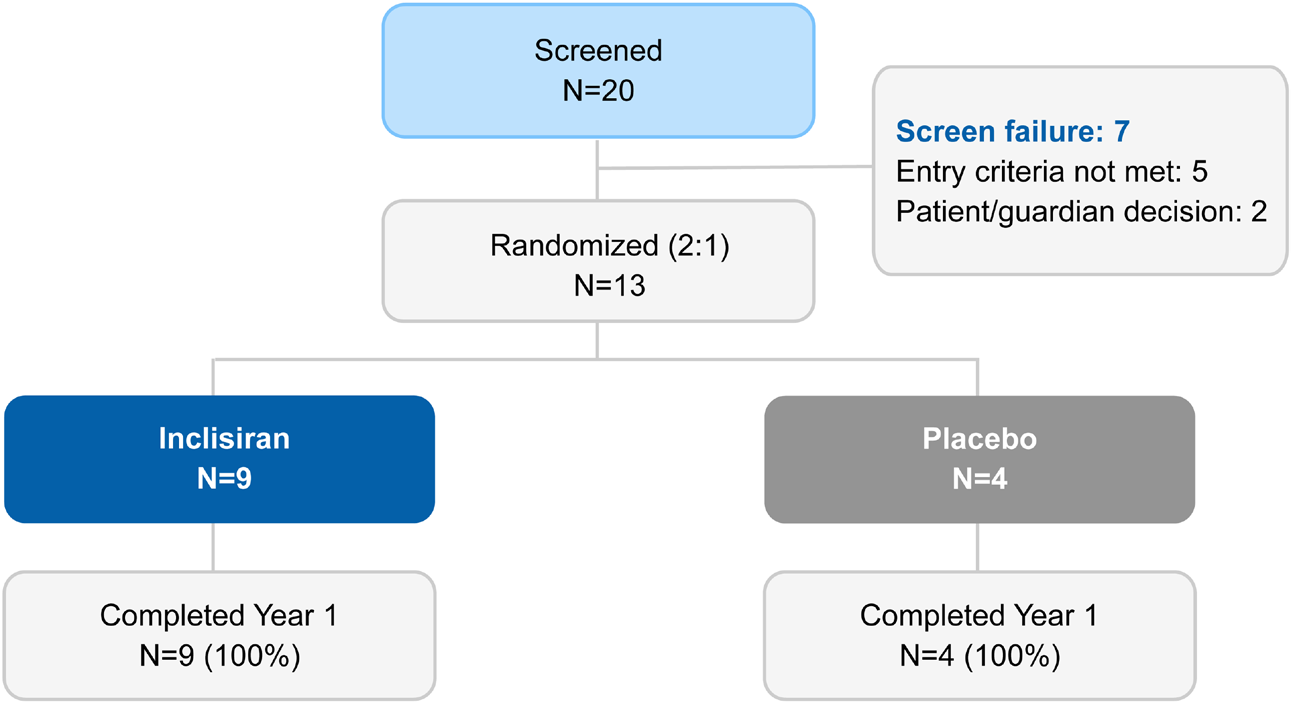
Patient disposition.

Of the 20 patients screened, 13 were enrolled, with 9 randomized to the inclisiran group and 4 to the placebo group. Of the 7 patients not enrolled, 5 were excluded for not meeting entry criteria (3 had an *LDLR* null/null genotype, and 2 had apheresis timing not in line with protocol requirements), and for the remaining 2, the patient or guardian declined to participate. All (100%) patients completed part 1 of the study (Figure 1) and received all 3 doses of the study medication. Patient demographic and clinical characteristics are summarized in Table 1. Mean (SD) patient age was 14.8 (1.9) years; 61.5% (n=8) were <15 years of age, 69.2% (n=9) were female, and 84.6% (n=11) were White. The mean (SD) baseline LDL-C level was 272 (111) mg/dL. All patients were receiving statins, and 84.6% (n=11) of patients were treated in addition with ezetimibe with or without other LLTs. One patient in the placebo arm was on LDL apheresis. Information on the other LLTs is provided in Table S2. No patients were administered anti-PCSK9 mAbs either during the trial or within 90 days before the screening visit in line with protocol requirements.

**Table 1.**
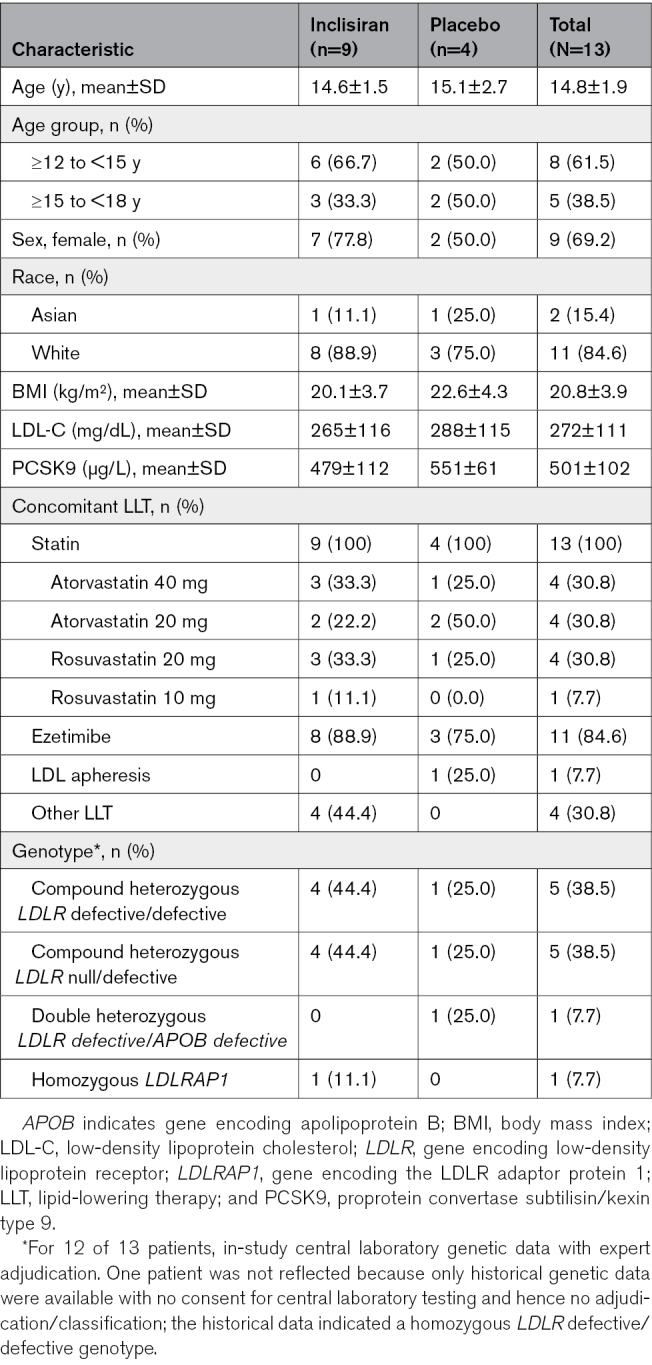
Baseline Demographic and Clinical Characteristics

A total of 12 (92.3%) patients underwent centralized genotyping (in addition to all 13 patients having a historical genetic diagnosis). Among these 12 patients, variants involving both *LDLR* alleles were present in 10 patients (5 patients had compound heterozygous *LDLR* defective/defective variants, and 5 had compound heterozygous *LDLR* null/defective variants), one was double heterozygous *LDLR* defective/*APOB* (n=1), and one was homozygous *LDLRAP1* (n=1). Details are provided in Table S3.

### Efficacy

**Figure 2.**
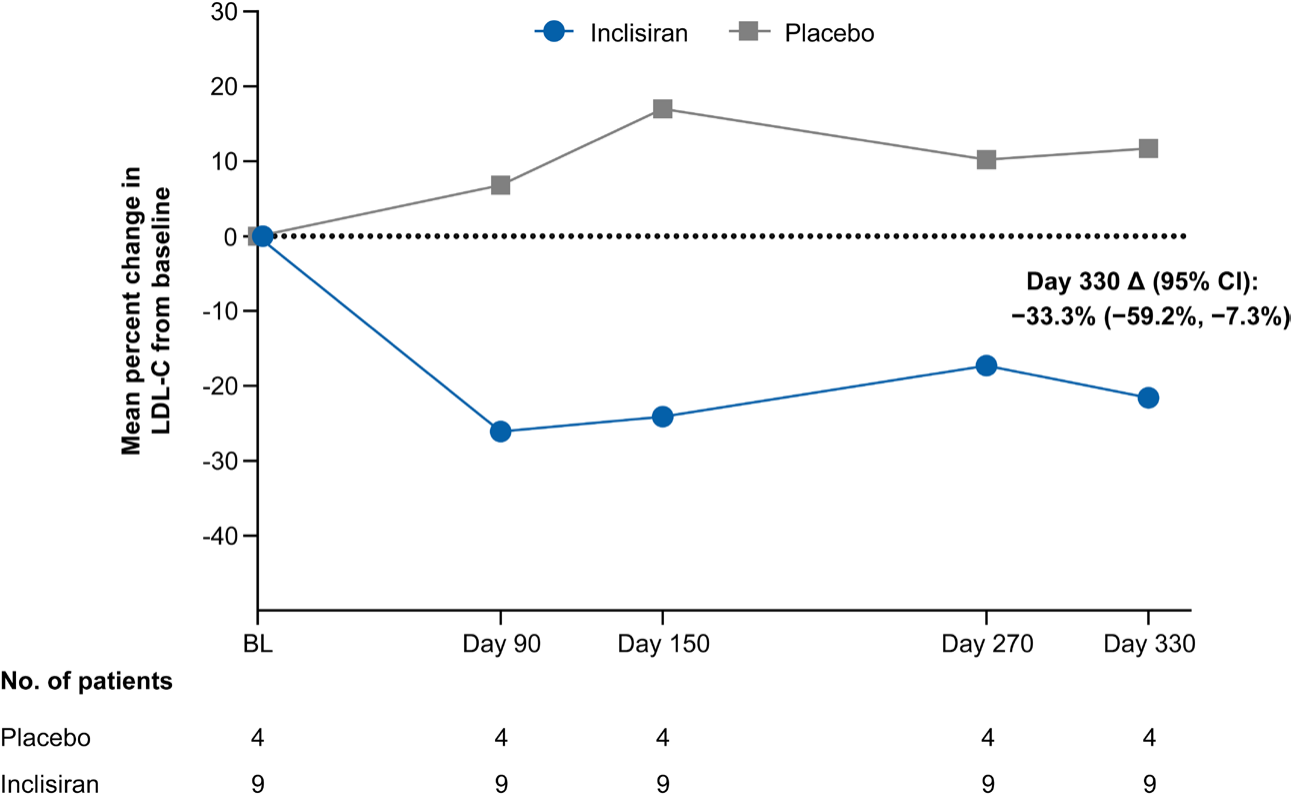
**Mean percentage change in LDL-C from baseline at days 90, 150, 270, and 330 (full analysis set).** Δ indicates mean between group-difference; BL, baseline; and LDL-C, low-density lipoprotein cholesterol.

The mean (SD) percentage change in LDL-C from baseline at day 330 was −21.6% (13.4%) in the inclisiran group and +11.7% (30.5%) in the placebo group, with a mean between-group difference of −33.3% (95% CI, −59.2% to −7.3%; Figure 2; Figure S1). Although formal hypothesis testing was not planned, the 95% CI indicates nominal statistical significance. Consistent results in the 2 treatment groups were observed using median percentage reductions in LDL-C. Inclisiran reduced LDL-C from the first assessment (at day 90), with reductions maintained through day 330 (Figure 2).

**Figure 3.**
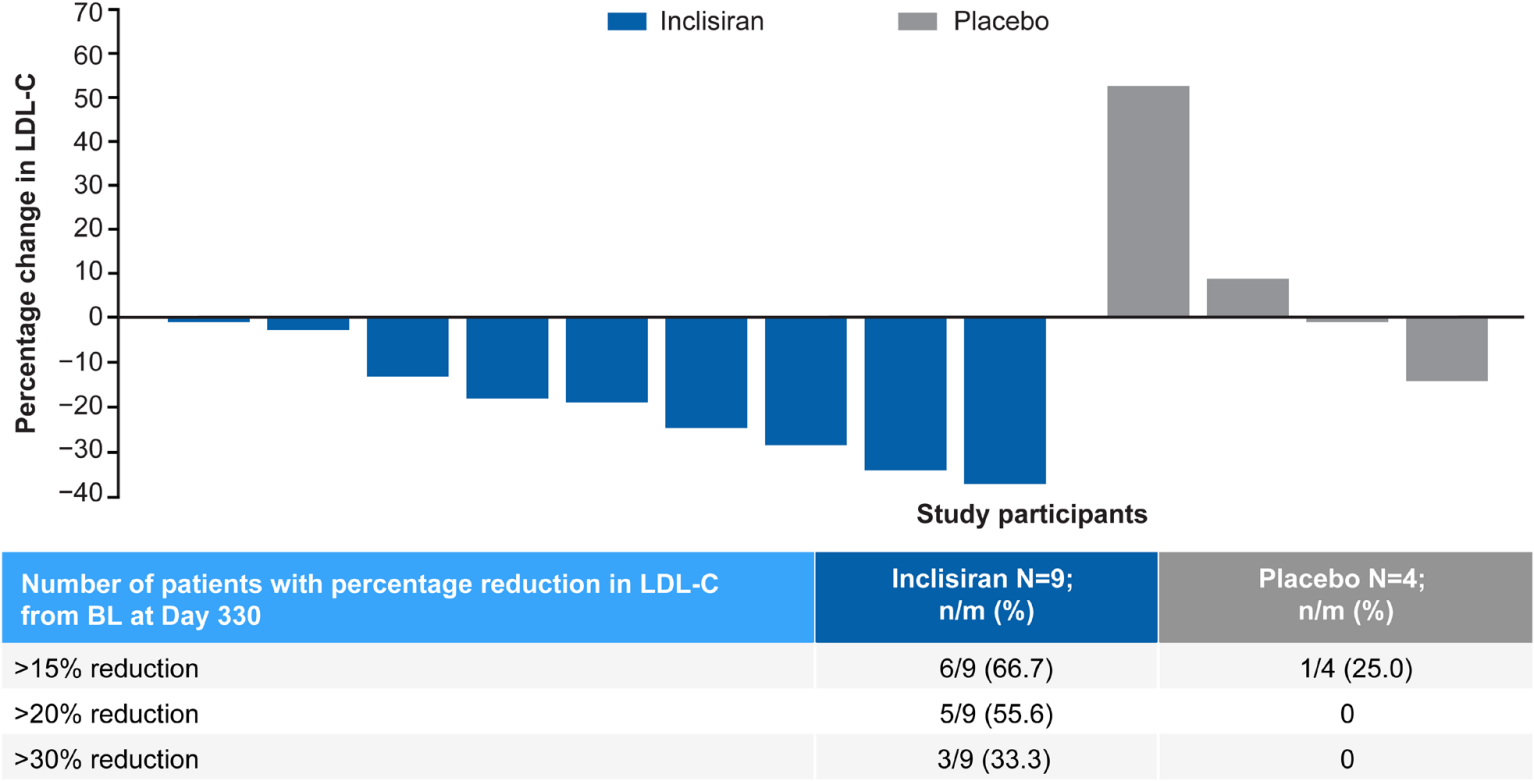
**Waterfall plot of percentage change in LDL-C level from baseline to day 330 (full analysis set).** BL indicates baseline; and LDL-C, low-density lipoprotein cholesterol.

A waterfall plot of percentage change in LDL-C levels from baseline to day 330 by individual patient responses is shown in Figure 3. Although of variable magnitude, all patients in the inclisiran group showed a decrease in LDL-C levels. In 6 of 9 (67%) inclisiran-treated patients, the LDL-C reduction was >15%, in 5 of 9 (56%) patients, it was >20%, and in 3 of 9 (33%) patients, it was >30%. The response in the placebo group was less consistent, with patients showing increases and decreases (1 patient >15%) in LDL-C levels.

The mean (SD) time-adjusted percentage change in LDL-C after day 90 up to day 330, defined as the average percentage change in LDL-C from baseline to days 150, 270, and 330, was −21.0% (15.1%) in the inclisiran group and +13.0% (41.9%) in the placebo group, with a between-group difference of −34.0% (95% CI, −67.5% to −0.4%), very similar to the result at day 330. The placebo-adjusted mean absolute change in LDL-C from baseline to day 330 was −74.6 mg/dL (95% CI, −150.9 mg/dL to 1.7 mg/dL), resulting from mean (SD) changes of −62.9 mg/dL (52.4 mg/dL) and +11.8 mg/dL (69.9 mg/dL) in the inclisiran and placebo groups, respectively. The absolute change in LDL-C from baseline to day 330 for each patient is shown in Figure S2. The placebo-adjusted mean percentage changes from baseline to day 330 in apoB, non-HDL cholesterol, and total cholesterol were −23.0%, −32.7%, and −27.8%, respectively (Table 2). On day 330, the mean percentage change in PCSK9 from baseline was −65.3% in the inclisiran group versus −5.1% in the placebo group (between-group difference, −60.2%; Table 2).

**Table 2.**
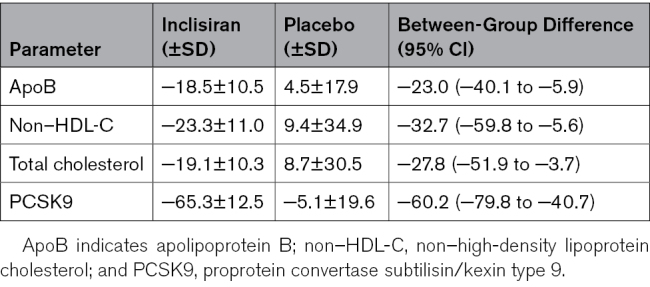
**Mean Percentage Change in Lipid Parameters and PCSK9 From Baseline to Day 330 (Full Analysis Set**)

A subgroup analysis by genotype, to evaluate the reduction in LDL-C according to underlying causal genetic variants of HoFH, showed consistent LDL-C lowering with inclisiran irrespective of the genotype. For the 2 main genotypes, compound heterozygous *LDLR* defective/defective and compound heterozygous *LDLR* null/defective, the mean (SD) percentage changes in LDL-C from baseline to day 330 with inclisiran were −20.5% (12.0%) [n=4; placebo: +9.0%, n=1] and −18.1% (14.4%) [n=4; placebo: −1.7%, n=1], respectively. The one patient in the inclisiran group with a homozygous *LDLRAP1* pathogenic variant showed a −39.7% reduction in LDL-C, whereas the one patient with a double heterozygous *LDLR*/*APOB* genotype in the placebo group showed an increase in LDL-C (+54.9%).

### Safety Analysis

A total of 7 of 9 (77.8%) inclisiran-treated patients and 1 of 4 (25.0%) placebo-treated patients experienced at least one treatment-emergent AE. The majority of reported AEs were mild. Moderate AEs were seen in 1 of 9 (11.1%) inclisiran-treated patients and 1 of 4 (25.0%) placebo-treated patients. No severe AEs were reported in either group. The most commonly reported AEs were COVID-19 (inclisiran group: 4 of 9 [44.4%] patients; placebo group: 1 of 4 [25%] patients), upper abdominal pain (2 of 9 [22.2%]; 0 of 4 [0%]), gastroenteritis (1 of 9 [11.1%]; 1 of 4 [25%]), injection site reaction (2 of 9 [22.2%]; 0 of 4 [0%]), and fever (2 of 9 [22.2%]; 0 of 4 [0%]). AEs related to study drug were reported in 2 of 9 (22.2%) inclisiran-treated patients and 0 of 4 (0%) placebo-treated patients. The study drug–related AEs were mild, transient injection site reactions, which resolved without sequelae and did not lead to study drug discontinuation. No treatment-emergent serious AEs and no discontinuations of the study drug because of AEs were reported in either treatment group. There were no deaths reported in the study.

No laboratory abnormalities of ALT (alanine transaminase)/AST (aspartate transaminase) >3× the upper limit of normal, bilirubin >2× the upper limit of normal, creatinine >2 mg/dL, and CK (creatine kinase) >5× the upper limit of normal occurred in either treatment group. There were no adverse effects seen with inclisiran treatment on growth (height, weight, and body mass index), pubertal development (as assessed using Tanner staging), and hormonal status (estradiol/testosterone, LH, FSH, cortisol, and dehydroepiandrosterone sulfate). No patients in either treatment group developed treatment-induced antidrug antibodies. No new safety signals were identified.

## Discussion

ORION-13 is the first study to evaluate the efficacy and safety of inclisiran in adolescents with HoFH and reports critical data on the use of inclisiran in a pediatric population. In the randomized, placebo-controlled, double-blind part 1 of this multicenter study, inclisiran was effective in lowering LDL-C in patients with HoFH and a minimum of LDLR residual activity who received maximally tolerated statin treatment (with or without other lipid-lowering therapies). Inclisiran was well tolerated, and the safety findings are consistent with previously reported studies in adults and the overall benign safety profile of inclisiran in various adult patient populations including ASCVD, ASCVD risk equivalent, HoFH, and heterozygous familial hypercholesterolemia.^20–22,25,26^

A unique feature of ORION-13 is that part 1 of the study had a randomized and controlled/double-blind design, allowing a more reliable assessment of the treatment effect of inclisiran, whereas previous trials in pediatric patients with HoFH have typically been uncontrolled.^8,10,27–29^ The placebo-controlled design was ethically justifiable because all patients entering the study were on optimal standard-of-care HoFH treatment and because the very severe HoFH *LDLR* null/null cases were excluded from the study as further discussed below.

The study demonstrated a robust, clinically relevant reduction in LDL-C with inclisiran (−21.6%) versus an increase in the placebo group (11.7%), resulting in a between-group difference at day 330 of −33.3% on top of standard-of-care treatment in this difficult-to-treat patient population. The LDL-C–lowering effect of inclisiran was accompanied by relevant improvements in other lipid parameters, including total cholesterol, non-HDL cholesterol, and apoB. Two-thirds of the patients in the inclisiran group reached a >15% LDL-C reduction, more than half had a >20% improvement in LDL-C levels, and one-third had a >30% LDL-C reduction. A >15% LDL-C reduction was described in the 2023 European Atherosclerosis Society consensus statement update on HoFH as being a clinically relevant improvement with a PCSK9-directed therapy, warranting treatment continuation after initiation in clinical practice.^2^

Lowering LDL-C levels is currently the main treatment goal in familial hypercholesterolemia and shows an approximately linear relationship with the incidence of ASCVD.^2,30^ Lowering LDL-C with PCSK9 inhibitors also significantly reduces ASCVD risk.^31,32^ Therefore, effective lowering of LDL-C by inclisiran, over time, can also be expected to decrease the risk of ASCVD, which is currently being evaluated in adults in two ongoing studies (NCT03705234 and NCT05030428).

In ORION-2, a small (n=4) pilot study on adult patients with HoFH, inclisiran treatment resulted in a mean percentage reduction in LDL-C from baseline at day 180 of −21.0%.^33,34^ However, in the phase 3 ORION-5 trial (n=56), inclisiran did not reduce LDL-C levels in the overall population of adult patients with HoFH despite substantial lowering of PCSK9 levels.^26^ Several confounding factors were identified that might have contributed to the observed overall result and were taken into account in the study design of the adolescent ORION-13 study. These factors included enrollment of a high number of subjects with *LDLR* null/null variants (27% in the inclisiran group) as well as the time point of apheresis administration relative to the LDL-C measurements, including at baseline, in the >35% apheresis-treated patients.^26^ In line with these considerations, a post hoc analysis, which was performed to evaluate the effect of inclisiran on LDL-C lowering excluding patients with apheresis treatment and the *LDLR* null/null variants (n=30), showed reductions in LDL-C ranging from −12.9% to −30.0%.^26^

Inclisiran in adolescent patients demonstrated consistent and clinically meaningful LDL-C lowering across the included genotypes (ie, compound heterozygous *LDLR* defective/defective, compound heterozygous *LDLR* null/defective, and homozygous *LDLRAP1* variants). Based on the LDLR-dependent mechanism of action of inclisiran,^19^ as described earlier, and the insights gained from the inclisiran study in adult HoFH, patients with an *LDLR* null/null genotype were excluded from the present study. Hence, the study supports the effectiveness of inclisiran in adolescents with HoFH who are predicted to have a minimum amount of *LDLR* residual activity (non-null), although we cannot exclude a minimal response in those with biallelic *LDLR* null/null variants.

Several trials have studied lipid-lowering therapies in children and adolescents with HoFH, showing variable results. Pediatric patients (10 to <18 years of age) with HoFH (based on a clinical or genetic diagnosis; all genotypes) were included in 3 single-arm, open-label multicenter studies investigating the PCSK9 mAb evolocumab.^27^ In the HAUSER-OLE study (HAUSER Open-Label Extension), the median percentage change in LDL-C from baseline to week 80 in pediatric patients with HoFH was −14.3% (n=12).^27^ In the TAUSSIG study (Trial Assessing Long-Term Use of PCSK9 Inhibition in Subjects With Genetic LDL Disorders), adolescent patients (n=14) showed a mean reduction in LDL-C of −10.6% at week 12 and −23.0% at week 48,^12^ and in RAMAN (The Safety and Tolerability of Reptha (evolocumab) in Indian Participants With Homozygous Familial Hypercholesterolemia), a single-country study in India, the mean change in LDL-C at week 12 was +1.0% in the 13 patients <18 years of age.^8^ In a pediatric (8 to <18 years of age; n=18) study in HoFH (genetic diagnosis excluding *LDLR* null/null but n=6 included) with the PCSK9 mAb alirocumab, the mean percentage change in LDL-C from baseline at week 12 was −4.1%; however, half of the patients reached a ≥15% reduction in LDL-C.^10^ In an open-label, single-arm study that included 14 adolescents with HoFH, evinacumab, an anti–angiopoietin-like protein 3 mAb requiring intravenous infusion every 4 weeks, reduced LDL-C by −55.4% at week 24.^29^ Evinacumab acts independently of LDLR function and will likely be most useful in severe *LDLR* null/null patients with HoFH. In the open-label APH-19 study in 43 pediatric patients with HoFH, lomitapide, an inhibitor of the microsomal triglyceride transfer protein associated with a risk of hepatoxicity and gastrointestinal adverse reactions,^2,35,36^ the mean change in LDL-C at week 24 was −53.5%.^28^

The strengths of part 1 of the ORION-13 study include its randomized, controlled, double-blind study design. This is contrary to previous clinical trials in adolescent HoFH populations, which have typically been open-label studies.^8,10,27–29^ A limitation of this study, influenced by the low prevalence of HoFH, is the small sample size (n=13) precluding formal statistical analysis.

### Conclusions

In the difficult-to-treat adolescent patient population with HoFH, inclisiran was effective in lowering LDL-C, showing a placebo-adjusted reduction of −33.3%, with 6 of 9 inclisiran-treated patients reaching a clinically relevant >15% improvement in LDL-C. Inclisiran was well tolerated, with a safety profile comparable to that seen in adults. The results of this study support inclisiran as a potentially useful addition for the treatment of adolescents with HoFH and a minimum of LDLR residual activity.

## ARTICLE INFORMATION

### Acknowledgments

The authors thank the adolescents and their families as well as the investigators and their staff for participating in this trial. The authors also thank Shreekant Sharma, MPharm (Novartis Healthcare Pvt Ltd India), and Tony Walsh, PhD (Novartis Ireland Ltd), for providing medical writing support, which was funded by Novartis Pharma AG, Basel, Switzerland, in accordance with Good Publication Practice (GPP3) guidelines (https://www.ismpp.org/gpp3), and Klaus Molle, PhD (Novartis Pharma AG, Switzerland), for critical review of the article.

### Sources of Funding

This study was initiated and funded by Novartis Pharma AG. The funder was also involved in the study design, data collection, analysis, and interpretation, and provided review and feedback on the article. The authors, including both academic members from the study steering committee and funder employees, maintained full editorial control over the article and provided final approval for all content. All authors had access to the data and contributed to the review and revision of article drafts. The authors vouch for the accuracy and completeness of the data and adherence of the trial to the protocol.

### Disclosures

A.W. reports research grants from Amgen, Esperion, Novartis, Regeneron, Sanofi, Silence Therapeutics, and Ultragenyx, and consulting fees from Chiesi, Novartis, and Ultragenyx. A.L.P. reports participation on the Novartis steering committee. R.A.H. reports consulting fees from Acasti, Aegerion, Akcea/Ionis, Amgen, Arrowhead, HLS Therapeutics, Medison, Novartis, Pfizer, Regeneron, Sanofi, and Ultragenyx. E.B. reports consulting fees from Aegerion, Akcea/Ionis, Amgen, Chiesi, Ipsen, Novartis, Pfizer, Sanofi, Servier, Viatris, and Ultragenyx. A.S. is an employee of Novartis and owns Novartis shares. A.L. and Y.W. are employees of Novartis. J.D. reports consulting fees from Novartis.

### Supplemental Material

Tables S1–S3

Figures S1 and S2

Consolidated Standards of Reporting Trials (CONSORT) Checklist

## Supplementary Material


