# Supplementary material for: Efficacy and Safety of Inclisiran in Adolescents With Genetically Confirmed Homozygous Familial Hypercholesterolemia: Results From the Double-Blind, Placebo-Controlled Part of the ORION-13 Randomized Trial
Source: Circulation. 2025 May 20;151(25):1758–66. doi: 10.1161/CIRCULATIONAHA.124.073233 (PMC12180692; doi:10.1161/CIRCULATIONAHA.124.073233)
Supplement: Supplementary file 1 [file cir-151-1758-s001.pdf]

SUPPLEMENTAL MATERIAL

Contents

Table S1: Study site and investigator details 2

Table S2. Reported concomitant lipid-lowering therapies other than statins, ezetimibe and LDL-C apheresis (reflected as "other" in Table 1) 3

Table S3. Patient genotype characteristics 4

Figure S1. Mean percentage change in LDL-C from baseline at Day 330 5

Figure S2. Absolute change in LDL-C from baseline to Day 330 by patient (full analysis set) 6

Supplemental CONSORT checklist 7

Table S1: Study site and investigator details

Country	PI name	Site	City
Canada	Jean Bergeron	Clinique des maladies lipidiques de Québec	Québec
France	Noel Peretti	Hopital Femme Mere Enfant	Bron/Lyon
Greece	Genovefa Kolovou	Metropolitan Hospital Cardiology Clinic	Piraeus
Lebanon	Hala Tfayli	American University of Beirut Medical Center	Beirut
Malaysia	Azriyanti Binti Anuar Zaini	University Malaya Medical Center Department of Pediatrics	Kuala Lumpur
Netherlands	Albert Wiegman	Amsterdam University Medical Center	Amsterdam
Türkiye	Sema Kalkan Ucar	Ege University Medical Faculty	Izmir
	Ayse Cigdem Aktuglu Zeybek	Istanbul University Cerrahpasa Faculty of Medicine	Istanbul
USA	Seth J. Baum	Excel Medical Clinical Trials LLC	Boca Rotan

PI, principal investigator; USA, United States of America.

Table S2. Reported concomitant lipid-lowering therapies other than statins, ezetimibe and LDL-C apheresis (reflected as “other” in Table 1)

Patient	Treatment	Lipid-lowering therapies
1	Inclisiran	Colesevelam and Niacin
2	Inclisiran	Colesevelam
3	Inclisiran	Omega-3 fatty acids
4	Inclisiran	Omega-3 fatty acids

Table S3. Patient genotype characteristics

Patient*	Age/Sex	Treatment	Variant type	Adjudicated genotype and functional status
1	16/F	Inclisiran	*LDLR* c.2043C>A/ *LDLR* c.761A>C	Compound heterozygous *LDLR* null/defective
2	14/F	Inclisiran	*LDLR* c.1747C>T/ *LDLR* c.340-344delTTTCG	Compound heterozygous *LDLR* null/defective
3	14/F	Inclisiran	*LDLRAP1* c71dupG/ *LDLRAP1* c71dupG	Homozygous *LDLRAP1*
4	16/F	Inclisiran	*LDLR* c.1747C>T/ *LDLR* c.1879G>A	Compound heterozygous *LDLR* defective/defective
5	13/F	Placebo	*LDLR* c.1747C>T/ *LDLR* c.1879G>A	Compound heterozygous *LDLR* defective/defective
6	16/F	Placebo	*LDLR* c.917C>T *APOB* c.10580G>A	Double heterozygous *LDLR* defective/*APOB* defective
7	13/F	Inclisiran	*LDLR* c.1359-1G>A/ *LDLR* c.1775G>A	Compound heterozygous *LDLR* null/defective
8	13/M	Inclisiran	*LDLR* c.517T>C/ *LDLR* c.858C>A	Compound heterozygous *LDLR* defective/defective
9	12/F	Inclisiran	*LDLR* c.1646G>A/ *LDLR* c.81C>G	Compound heterozygous *LDLR* defective/defective
10	12/M	Placebo	*LDLR* deletion of exon 1/ *LDLR* c.259T>G	Compound heterozygous *LDLR* null/defective
11	13/M	Inclisiran	*LDLR* c.2043C>A/ *LDLR* c.761A>C	Compound heterozygous *LDLR* null/defective
12	16/F	Inclisiran	*LDLR* c.514G>A/ *LDLR* c.530C>T	Compound heterozygous *LDLR* defective/defective

*Presented data for 12/13 patients with in-study central laboratory genetic data and expert adjudication; one patient not reflected as only historical genetic data available with no consent for central laboratory testing and hence no adjudication/classification; the historical data indicated a homozygous *LDLR* defective/defective genotype (variant type: *LDLR* c.1567G>A / *LDLR* c.1567G>A). F, female; M, male.

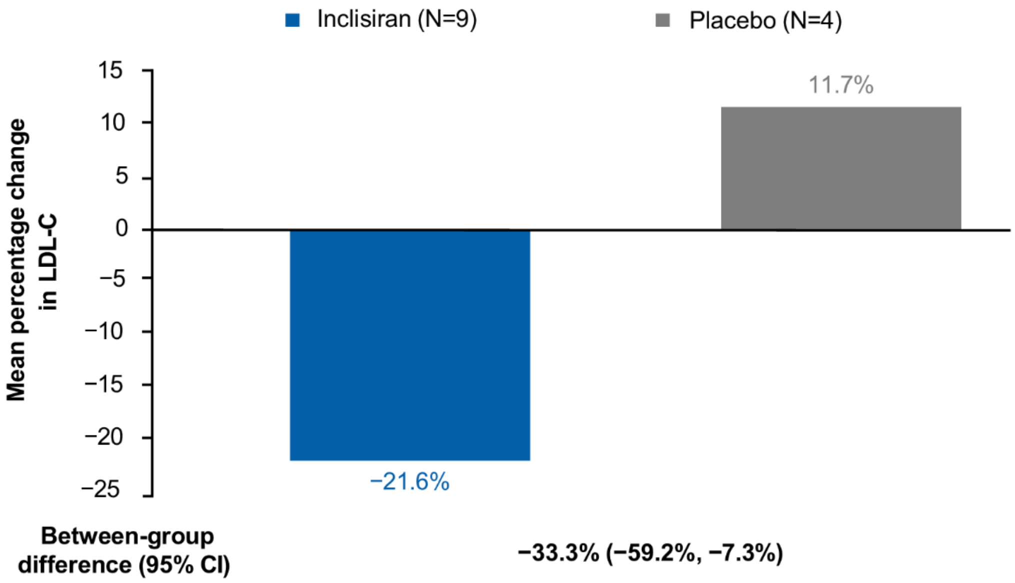

Figure S1. Mean percentage change in LDL-C from baseline at Day 330

CI, confidence interval; LDL-C, low-density lipoprotein cholesterol.

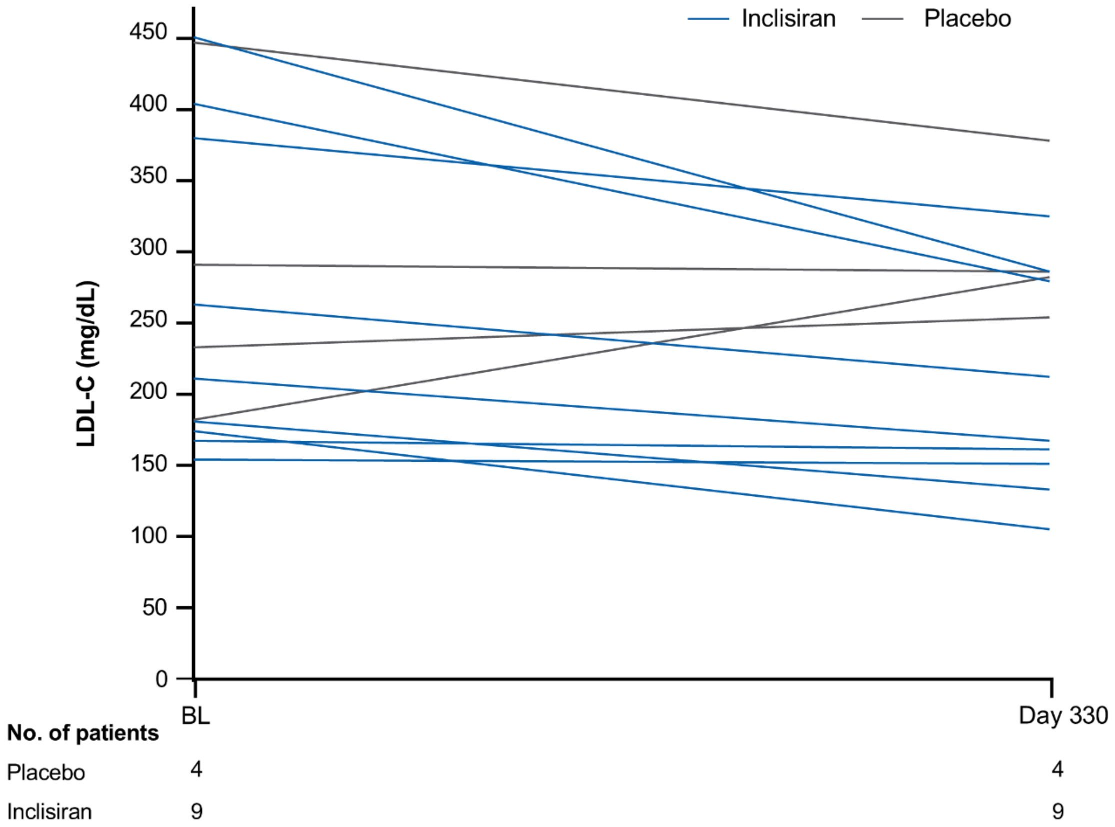

Figure S2. Absolute change in LDL-C from baseline to Day 330 by patient (full analysis set)

BL, baseline; LDL-C, low density lipoprotein cholesterol.

Supplemental CONSORT checklist

		Reporting Item	Page Number
**Title and Abstract**			
Title	#1a	Identification as a randomized trial in the title.	1
Abstract	#1b	Structured summary of trial design, methods, results, and conclusions	3, 4
**Introduction**			
Background and objectives	#2a	Scientific background and explanation of rationale	6, 7
Background and objectives	#2b	Specific objectives or hypothesis	7
**Methods**			
Trial design	#3a	Description of trial design (such as parallel, factorial) including allocation ratio.	7, 8
Trial design	#3b	Important changes to methods after trial commencement (such as eligibility criteria), with reasons	NA
Participants	#4a	Eligibility criteria for participants	8
Participants	#4b	Settings and locations where the data were collected	7
Interventions	#5	The experimental and control interventions for each group with sufficient details to allow replication, including how and when they were actually administered	8
Outcomes	#6a	Completely defined prespecified primary and secondary outcome measures,	8-10

		including how and when they were assessed	
Outcomes	#6b	Any changes to trial outcomes after the trial commenced, with reasons	NA
Sample size	#7a	How sample size was determined.	9
Sample size	#7b	When applicable, explanation of any interim analyses and stopping guidelines	NA
Randomization - Sequence generation	#8a	Method used to generate the random allocation sequence.	
8			
Randomization - Sequence generation	#8b	Type of randomization; details of any restriction (such as blocking and block size)	
8			
Randomization - Allocation concealment mechanism	#9	Mechanism used to implement the random allocation sequence (such as sequentially numbered containers), describing any steps taken to conceal the sequence until interventions were assigned	8
Randomization - Implementation	#10	Who generated the allocation sequence, who enrolled participants, and who assigned participants to interventions	8
Blinding	#11a	If done, who was blinded after assignment to interventions (for example, participants, care providers, those assessing outcomes) and how.	7 (Ref#23)
Blinding	#11b	If relevant, description of the similarity of interventions	NA
Statistical methods	#12a	Statistical methods used to compare groups for primary and secondary outcomes	9-10

Statistical methods	#12b	Methods for additional analyses, such as subgroup analyses and adjusted analyses	NA
**Results**			
Participant flow diagram (strongly recommended)	#13a	For each group, the numbers of participants who were randomly assigned, received intended treatment, and were analysed for the primary outcome	11, 19
Participant flow	#13b	For each group, losses and exclusions after randomization, together with reason	11, 19
Recruitment	#14a	Dates defining the periods of recruitment and follow-up	4 (CT.gov link)
Recruitment	#14b	Why the trial ended or was stopped	NA
Baseline data	#15	A table showing baseline demographic and clinical characteristics for each group	22
Numbers analysed	#16	For each group, number of participants (denominator) included in each analysis and whether the analysis was by original assigned groups	12, 13, 20, 21, 23 (Supplement: 5, 6)
Outcomes and estimation	#17a	For each primary and secondary outcome, results for each group, and the estimated effect size and its precision (such as 95% confidence interval)	12, 13
Outcomes and estimation	#17b	For binary outcomes, presentation of both absolute and relative effect sizes is recommended	NA
Ancillary analyses	#18	Results of any other analyses performed, including subgroup analyses and adjusted analyses, distinguishing pre-specified from exploratory	13
Harms	#19	All important harms or unintended effects in each group (For specific guidance see CONSORT for harms)	13, 14

Discussion

Limitations	#20	Trial limitations, addressing sources of potential bias, imprecision, and, if relevant, multiplicity of analyses	17
Generalisability	#21	Generalisability (external validity, applicability) of the trial findings	5, 17
Interpretation	#22	Interpretation consistent with results, balancing benefits and harms, and considering other relevant evidence	17
Registration	#23	Registration number and name of trial registry	4

Other information

Interpretation	#22	Interpretation consistent with results, balancing benefits and harms, and considering other relevant evidence	17
Registration	#23	Registration number and name of trial registry	4
Protocol	#24	Where the full trial protocol can be accessed, if available	10
Funding	#25	Sources of funding and other support (such as supply of drugs), role of funders	18
